# Return to work or leaving work? Differences of return to work between breast cancer patients and the general population and determinants of return to work

**DOI:** 10.1007/s00520-025-09364-2

**Published:** 2025-03-22

**Authors:** Siegfried Geyer, Stefanie Sperlich, Eranda Sahiti, Dorothee Noeres

**Affiliations:** Department of Medical Sociology, Hannover Medical School, Carl-Neuberg-Strasse 1, 30623 Hannover, Germany

**Keywords:** Breast cancer, Return to work, Multicentric study, Longitudinal study, Observational study

## Abstract

**Purpose:**

It was examined whether employment among breast cancer survivors was lower than in the general population 4 to 6 years after surgery. We also examined whether disease severity, post-surgical treatment, social, and workplace characteristics have effects on employment as primary outcome, and whether the distance from surgery to observation may determine employment.

**Methods:**

We performed a multicentric observational study with four survey waves. Data were collected based on mailed surveys and patient records. Patients were up to 63 years old at entry with TNM-tumour stages T0 to TIV. Comparisons with the general population were performed by drawing controls from the German Socio-Economic Panel.

**Results:**

*N* = 372 breast cancer survivors participated in all surveys (= 82.2% of the initial sample). Their rate of occupationally active women was lower than in the general population (OR_patients_ = 0.59; 95% CI = 0.42–0.84; *p* < 0.01). Among patients, tumour stage had no effects on employment 12 months after surgery; 4–6 years later, this was the case only among patients with the most unfavourable tumour stage (OR = 0.16; *p* = 0.01; 95% CI = 0.04–0.58). Antihormone therapy was unrelated with employment (OR = 0.80; *p* = 0.27; 95% CI = 0.54–1.19); inpatient rehabilitation was negatively associated at 12 months after surgery (OR = 0.47; *p* = 0.02; 95% CI = 0.25–0.89) and unrelated at the last survey wave (OR = 0.95; *p* = 0.86; 95% CI = 0.55–1.64). Compared with the lowest level of occupational autonomy, it was unrelated with employment 12 months after surgery (OR = 0.79; *p* = 0.75; 95% CI = 0.18–4.41), but for the highest level of autonomy, it had significant effects 4 to 6 years later (OR = 4.56; *p* = 0.04; 95% CI = 1.10–18.81). Effort-reward imbalance as a continuously scaled indicator of pre-surgery occupational distress was significantly associated with return to work 12 months after surgery (OR = 0.13; *p* < 0.01; 95% CI = 0.06–0.31), but it had no effect at the last survey wave (OR = 0.64; *p* = 0.31; 95% CI = 0.28–1.50). One year after surgery, education at higher levels had no significant effects on return to work (OR = 1.30; *p* = 0.57; 95% CI = 0.56–3.00 for the highest level compared with the lowest one), only at the last measurement marked differences by education emerged (OR = 2.23; *p* = 0.03; 95% CI = 1.08–4.63).

**Conclusion:**

Temporal distance between surgery and survey wave determines whether potentially influencing factors have effects. Disease severity and post-surgical treatment were unrelated to employment. Whether work-related and socio-demographic factors are determining employment depends on the date of measurement.

## Introduction

A substantial number of breast cancers are occurring below retirement age. Between 2017 and 2021, the median age at diagnosis was 63 years in the USA [31]; in Europe, the figures are similar [39]; and in Germany, the median age at diagnosis was 65 years in 2019/2020 [26]. At the date of diagnosis, the majority of women is in paid employment or self-employed, either working full- or part-time. In 2017, 75.2% of women between 20 and 64 years of age were economically active [33]. Thus, breast cancer survivors have to consider whether or when to return to work after the main treatment having ended. For employed women with breast cancer, return to work is an important step back to normal, even if disease-related aftermaths and impairments are persisting [42]. In the literature, a multitude of factors affecting return to work are discussed. This includes disease-related, personal, employment-, socio-demographic, and contextual factors [18, 25, 35, 38, 43].

The most obvious obstacles for return to work are disease severity and disease-related comorbidity that were reported in a prospective study at about 23 months after diagnoses of different types of cancers with 59.5% breast cancer patients [20]. In a German study, 40 weeks after breast cancer diagnosis, patients with advanced tumour stages were less likely to return to work than patients with less severe stages [13]. Similar findings were reported in a study that covered 5 years after diagnosis [30]. Another longitudinal study examined effects of disease severity (depicted as tumour stage) and side effects of treatment on return to work 6 years after diagnosis [22]. Tumour stage also turned out as unrelated with return to work in a French study that covered 3 years after diagnosis [28].

The results of studies on effects of the type of post-surgery cancer treatment (chemotherapy, radiation therapy, hormonal treatment) are inconsistent, and this mainly applies to differences of study settings in terms of time frame and the selection of patients. In a large study covering different entities, the type of aftertreatment did not predict return to work [1]. In a study with 135 disease-free breast cancer survivors, hormone therapy was associated with continuous participation in the workforce [30]. The severity of side effects of treatment was not predictive for return to work, and this applied irrespective whether an observation period of 1 or 6 years after breast cancer surgery was used [22]. Types of aftertreatment have also been mentioned in review papers discussing return to work [14, 35].

Inpatient oncological rehabilitation is a distinguishing mark of the German health care system directed towards a rapid return to work and to everyday life and to prevent early retirement. For employed patients, it is covered by the pension insurance. It is directed towards the minimisation of physical and psycho-social consequences of a malignant disease by offering specific medical and psychological therapies and counselling in inpatient or outpatient settings (https://www.deutsche-rentenversicherung.de/DRV/EN/Home/home_node.html). In a recent study, it was reported that 71.8% of breast cancer survivors had made use of at least one rehabilitation measure [12]. Inpatient rehabilitation turned out to be associated with lower rates of return to work after 1 year following surgery, but after 6 years, the effect was no longer present [22]. In another study, oncological inpatient rehabilitation was associated with a reduced chance of return to work covering an observation period of 40 weeks after surgery [13]; thus, the empirical findings are not consistent in that respect.

Socio-demographic and contextual factors refer to the social dimension of return to work. Indicators of socio-economic differentiation have been reported to having effects, although also not always consistent. A German study reported higher rates of return to work with increasing educational level [30, 45]. Education-related differences were also reported in another study, with the first one covering 1 and 6 years after surgery [22]. Contrary to expectation, compared with the lowest educational level, return to work was higher at the intermediate level, but it was lower if breast cancer patients reached the highest educational level [13]. This non-linear effect was reported in a study with breast cancer patients who underwent oncological rehabilitation. In contrast, for the material side of social differentiation, the rates of patients increased with rising income [20].

Taken together, there is evidence that social and occupation-related factors have effects on return to work. The studies published so far and review papers [14, 35, 43] had discussed a large number of factors, they had depicted different lengths of time periods, and no comparable control groups have been drawn for comparing patients and the general population. A main issue of studies published so far refers to different types of factors that may determine return to work. Having knowledge about these factors does not imply that it is known whether or to what extent return to work in breast cancer patients is different from the general population. In order to estimate this difference, control groups are needed. Only a few studies are available that used comparison groups. An Italian study used self-assignment of controls; thus, leaving it open whether selection effects may have occurred [18]. Covering the years 2000 to 2005, a population-based study from the Netherlands compared breast cancer patients with controls of the same age and reported that patients were more likely to losing paid employment, and patients with a worse diagnosis had an increased risk of receiving disability benefits five to 10 years after diagnosis [24]. In a registry-based interview study, *N* = 445 formerly employed breast cancer survivors were compared with a control sample using propensity score matching. It turned out that patients were less likely than controls to being employed within 6 months after diagnosis. These findings have however to be interpreted against the backdrop of unfinished aftertreatments [2]. A German study started in 2002/2004 what may have produced some different results than nowadays [22]. According to the German Federal Statistical Office in the western federal states of Germany, 59.1% of women earned an own income in the year 2002, and this figure rose to 71.1% in 2017 [4].

Beyond the reported findings, patients’ decisions to return to work or not are framed by the social security regulations of countries, thus leading to between-country differences of the rates of cancer survivors who return to work [29]. The German social security system permits patients with cancer who do not have reached retirement age to claim retirement benefits for reduction in earning capacity if they are able to work at least 3 up to 6 h per day. In case of disease severity making employment impossible and oncological rehabilitation not being successful, patients may also apply for permanent retirement due to reduction in earning capacity.

Against the backdrop of the considerations above, we designed a longitudinal multicentric observational study on return to work of economically active women after surgery of primary breast cancer with repeated measurements. The primary outcome of our study was employment status. The maximum age at entry was 63 years. The following research questions will be dealt with:

1. Are the rates of economically active breast cancer patients 4–6 years after surgery different from the general population? This refers to the comparison of patients with a control group.
2. Do disease severity and type of post-surgical treatment have effects on the return to work of breast cancer patients?
3. Do social structural and workplace characteristics have effects on return to work in breast cancer survivors?
4. Do the effects obtained under (2) and (3) differ according to the distance between primary surgery of breast cancer and assessment?

## Methods

### Study setting

The patient population was drawn within the framework of a multicentric longitudinal study that was performed in the federal state of Lower Saxony, Germany. Ten certified breast cancer centres and a specialised outpatient practice covered the larger Hannover area and the countryside. The full list of participating clinics was published in an earlier paper [23]. The study combined standardised and qualitative interview-generated data, information on the utilisation of inpatient rehabilitation measures, and detailed medical information on disease severity and on employment/earning status. The data collection was performed by means of mail surveys. Before applied in the four studies, all questionnaires were tested for length using standard pretests, and the comprehensibility was verified by means of cognitive testing [44].

### Patients

Breast cancer patients were contacted consecutively if they met the following inclusion criteria: first diagnosis of invasive primary breast cancer with tumour stages T0 to T3, age 63 years or younger at entry into the study, and economically active (employed or self-employed) at the date of diagnosis. Patients were approached by the treating physicians in hospital, by a study nurse or in writing. In case of having given written informed consent, a questionnaire was sent by mail, accompanied by another written invitation by the respective director of the clinic and by the research team. All invitation letters had the original signature of the director of the clinic where patients were admitted and treated, and after the initial invitation, two reminders were sent to improve study participation. Once having obtained written consent to participate, further study-related contacts were made by the research group. Follow-up questionnaires were also sent by mail. This was done in agreement with the methodological recommendations of the survey literature for improving response in mail surveys [6, 7, 41]. The main years when the baseline measurement took place were 2017 and 2018 (only one interview was performed in 2016), and in 2022 and 2023, the last follow-ups took place. The following analyses are based on the data of patients and controls who participated in the first as well as in the last survey wave.

Our study was performed in accordance with the ethical standards as laid down in the 1964 Declaration of Helsinki and its later amendments or comparable ethical standards. It was approved by the ethics committee of Hanover Medical School under number 2973–2015. Reporting of data is performed in accordance with the STROBE guidelines [34]. Further details can be found in a recent companion publication [36].

### Control group

Effects of breast cancer (or the disease considered) on social outcomes can only be interpreted properly if patients with breast cancer (or the disease considered) are compared with individuals from the same population who do not have cancer (or the disease considered in a given study). In our case, we designed our study in the way that a maximum of variables between patients and the general population were identical with the German Socio-Economic Panel (SOEP). The SOEP is a longitudinal survey project set up in 1984 for monitoring social and economic developments in Germany [9]. The data are collected annually under the supervision of the German Institute for Economic Research (Deutsches Institut für Wirtschaftsforschung-DIW). SOEP-data are available for academic and research purposes. Thus, the data of persons making up our control group from the SOEP were not collected as part of the project, and the data were already collected and drawn from the SOEP.

Individuals making up the control group were drawn with our first survey wave as point of departure. As in the patient sample, only employed women were included at entry, and changes that occurred between the first and the last survey wave included were recorded as endpoints. Then for every patient, employed women who belonged to the same *age* group were drawn from the general population data, and who had the same *occupational level*. As more than one potential individual was eligible to be assigned to the control group, random sampling was used for drawing controls. As for patients, controls were eligible only if they participated in both waves of the SOEP. In our case, drawing a control group turned out as complicated because the last follow-up fell into the time of the corona-pandemic (2022/2023); thus, biased samples were to be expected as participation in the population survey might have been affected by selection effects [15, 16]. As for both groups the lengths of follow-up periods had to be equal, we shifted the time period of controls backwards in order to avoid bias. Thus, it was decided that for the first study wave (2017/2018), controls were drawn from SOEP-survey waves 2014/2015, and for the last follow-up of patients, controls were drawn from the surveys 2019/2020.

### Matching patients and controls

Patients and controls were matched at a 1:2 ratio using *age* and *educational level* as combined criteria for pairing (see Table 1 for the categories of the two variables). Matching was performed multivariately, i.e. an employed cancer survivor at a defined age (i.e. 45 years) and a defined educational level (i.e. 10 years of school education) was matched with a woman from the general population with the same combination of characteristics. If more than two controls with a defined combination were found, the assignment to the control group was performed at random. Age as matching criterion was used because it is a risk factor for the disease, it is an indicator for a similar position in the life course and in the occupational career. Education was used as matching criterion because it is an indicator for occupational level that can be attained, and it is also related with the likelihood of leaving the job prematurely [22]. The matching process was successful, and all breast cancer cases were matched with at least two individuals from the general population. The supplementary STATA-module OPTMATCH2 was used for matching cases and controls [11, 17].

**Table 1:** Distributions of the variables used for pairing cases (*n* = 372) and controls (*n* = 744) and of variables used the regression analyses

	**Variables used for drawing the control group**		
	Breast cancer patients (*N* = 372)	SOEP-population sample (*N* = 744)	%
**Age group at T0** 24 to 27 years 28 to 31 years 32 to 35 years 36 to 39 years 40 to 43 years 44 to 47 years 48 to 51 years 52 to 55 years 56 to 59 years 60 to 63 years	3 3 10 13 27 43 84 87 68 34	6 9 24 27 55 84 166 173 136 64	0.81% 0.81% 3.32% 3.58% 7.35% 11.38% 22.22% 23.48% 18.28% 8.78
Educational level None, not completed 8/9 years of schooling 10 years of schooling 12/13 years of schooling Other degree Information missing	2 44 148 166 6 6	4 88 296 332 12 12	0.54% 11.38% 39.78% 44.62% 1.61% 1.61%
Tumour stage at surgery/TNM	**Breast cancer patients (*n*/%)**		
	Antihormne therapy/t2	Antihormone therapy at t3	Occupational autonomy at T0
0/I 228/61.13% II 118/31.64% III 25/6.70% IV 2/0.54%	Yes 232/62.2% No 141/37.8%	Yes 190/51.08% No 182/48.92%	1 low level 29/7.92% 2 some autonomy 82/22.40% 3 intermediate level 157/42.90% 4 elevated level 78/21.31% 5 high level 20/5.46% Missing 6

### Variables used in the analyses

In an empirical study, not all factors discussed in reviews and theoretical papers can be considered as the sample size of study populations usually does not permit to analyse too many of them. Nevertheless, we wanted to include aspects of disease severity, breast cancer treatment, and social/work-related factors.

Disease severity was classified according to TNM-related tumour size [3] based on the result of the histologic analysis. The following classifications were used: T0, no primary tumour can be detected after adjuvant chemotherapy; T1, tumour with a maximum extent of 2 cm; T2, tumour with an extent of more than 2 up to 5 cm; T3, tumour with an extent of more than 5 cm; T4, tumour that has grown into the chest wall or into the skin. Due to small case numbers, in the regression analyses, T-stages T0 and T1 were counted together. As a second indicator of disease severity, type of surgery was used. In the analyses, it was coded as ‘yes’ or ‘no’. Both were obtained from patient records assessed at baseline. More details in this part of data collection have been published in an earlier report [36].

Breast cancer treatment after surgery was depicted by two indicators. We included *antihormone* therapy because patients may have used it for years after primary therapy. It has side effects that may impair everyday functioning including decisions to return to work. As more than one therapeutic scheme was applied, this variable was coded in a ‘yes’ or ‘no’-format. We also considered inpatient *oncologic rehabilitation* because it is routinely offered for facilitating return to work, but the final decision whether to use it is left up to patients. The utilisation of oncologic rehabilitation was also coded as ‘yes’ or ‘no’.

Employment level referred to full- or part-time employment, classified separately for each survey wave. As all women with breast cancer were employed when receiving their diagnosis, employment levels at follow-up were differentiated by *employed/self-employed* (returned to work), *retired*, officially registered as unemployed (i.e. receiving unemployment benefits), and *not employed* (women without an own source of income). All categories were mutually exclusive. This information was asked at every survey follow-up, but for the comparisons with the general population, only data from the last survey wave were used.

#### Social structural and work-related factors

Education, occupational position, and income as the three most frequently used indicators of social differentiation were assessed. Finally, only education was used for drawing the control group and as a structural indicator for return to work in the regression analyses. According to the German school system, education was coded as ‘no degree’, ‘8/9 years of schooling’, ‘10 years’, ‘12/13 years’, or ‘other degree’.

Work-related indicators can be assumed to be better predictors than more distant and general indicators of social differentiation. Thus, we used work-related autonomy as an indicator for working conditions, scaled at five steps from ‘none’ to ‘high autonomy’. Finally, effort-reward imbalance (ERI) as a well-proven indicator for the subjective assessment of the balance between individual achievement and returns at work [32]. ERI is a continuously scaled variable with higher scores indicating a more negatively perceived work balance.

Age had to be considered as it has effects on the likelihood of a breast cancer diagnosis, on exit from employment, on individual considerations of patients whether to return to work or to retire prematurely, and it indicates regular retirement age. For the matching procedure, age was divided into 10 age groups (see Table 1); in the regression analyses, age was used in metric scaling.

### Statistics

Changes of employment status of patients and controls from the beginning of the study to follow-up were displayed in graphical mode using a *Sankey-graphic* that was performed using a supplementary module [21]. Then the graphic produced by the Sankey-module was completed by transferring it to MS-Power Point. In our case, all patients and all individuals from the control group were employed at the first observation, and the graphic depicts the static (i.e. continuously employed/self-employed) cases as well as changes towards unemployed, retired, and to not employed individuals at the final assessment (T3).

Frequencies of employment levels as dependent on survey wave of patients are depicted by cross-tabulation. In addition, effect sizes for being employed as compared to all other categories of employment status were estimated using logistic regression with bootstrap-based standard errors (50 replications), and effect sizes are depicted as odds ratios. Logistic regression was also used for estimating effects of predictors on employment for two time periods after surgery, i.e. after 12 months and 4–6 years. All statistical analyses were performed using STATA 16.1 [37].

## Results

A total of 372 patients participated in the first and in the last survey with complete datasets, and 744 controls could be drawn from the SOEP-data (Table 1). The first follow-up (T1) interview took place 6 months after the first one, the second one (t2) followed 12 months later, and the third follow-up (t3) was performed 4–6 (mean = 5.2) years later. The patient population at baseline (T0) consisted of *n* = 454 employed breast cancer patients interviewed in the first weeks after surgery. This corresponds to a response rate of 80.7% of those who had been invited to participate. At the second wave (T1), 94.5% of those who participated in the preceding wave (T0) responded, and 95.6% of them participated in the third wave (T2). At the fourth one (T3), 90.7% of the women from the T2-wave responded, corresponding to 82.2% of the initial study population. The mean age of patients in the analyses below at entry was 50.58 (Sd = 7.27) years. Out of the who gave consent to participate, 23 had died at the end of the final survey wave. Breast cancer survivors who changed their residence during the study period remained in the study sample as contact information was available. No data from the 108 patients were available who were contacted initially and who did not want to participate. An individual’s refusal means that no part of her data must be used. In contrast to refusals at entry into the study, it was possible to qualify dropouts or nonresponse during the study. Against the backdrop of high response rates, it turned out that tumour size as indicator of disease severity did not separate responders from non-responders. Breast cancer survivors with endocrine therapy were more likely to participate than those without. From the work-related predictors, higher degrees of job autonomy led to lower response rates at the end of the study.

### Changes of employment/self-employment to another status from the first to the last survey wave

**Fig. 1:**
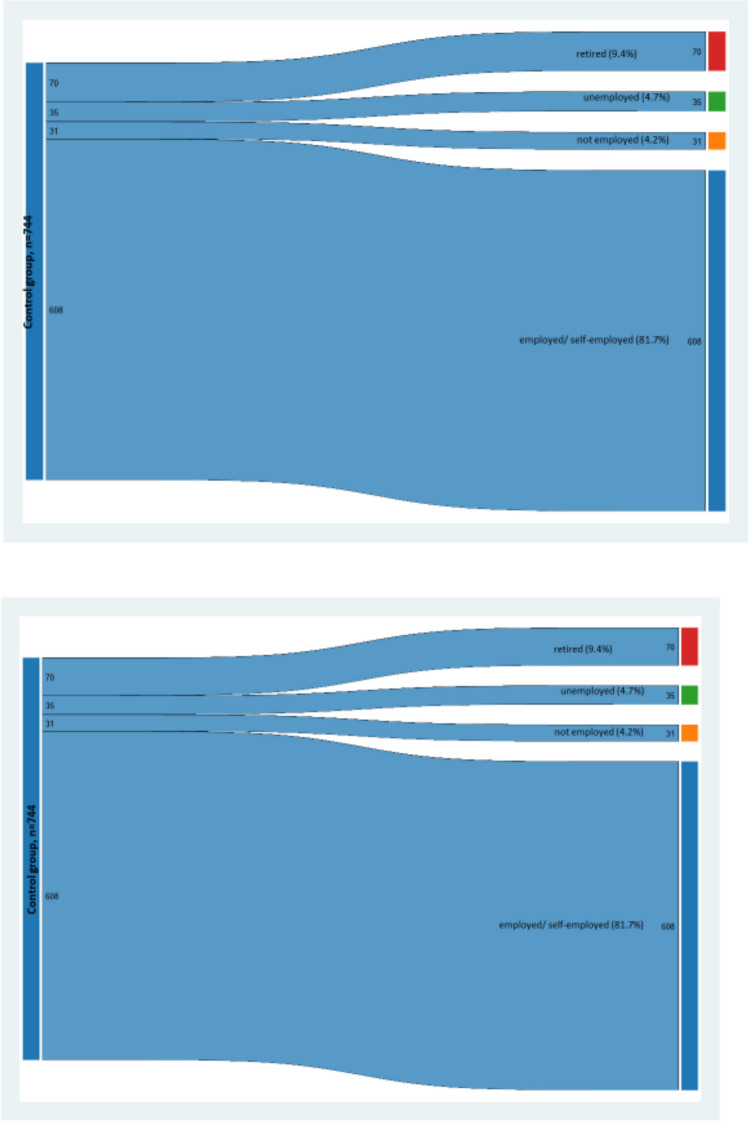
Changes from employment at the first survey wave to retired (red), not employed (orange), unemployed (green) at the last survey wave for breast cancer patients (upper graph) and for the control persons from the general population (lower graph)

Starting with full participation in the labour force at the first survey wave, Fig. 1 indicates that 5 years after surgery among patients, the rate of employed women was lower than among women from the general population. Against the backdrop of the same age structure, a higher proportion of patients had retired at the second survey wave. The rate of unemployed women with breast cancer was smaller than among controls.

In order to examine employment differences between patients and controls, a logistic regression with group membership (breast cancer survivors and SOEP-controls) as independent and employment as dependent variable was performed. Due to the matching process, the main variables assumed to have effects on employment were identical in both samples. Thus, the regression analysis was based on a parsimonious model.

The odds ratio (OR) indicates a substantial difference between groups with patients having a lower employment rate than controls (Table 2).

**Table 2:** Effects (in terms of odd ratios (OR)) of group membership on employment for the last survey wave after bootstrapping with *n* = 30 samples

	**OR**	***p***	**95% CI**
Employment SOEP-controls Breast cancer survivors	1 (ref.) 0.59	– 0.03	– 0.42–0.87
Constant	4.47	< 0.001	3.66–5.46

As displayed in Table 3, the lowest employment rates occurred in the first 6 months after surgery, employment was highest at 12 months later, and decreased again when the last assessment had taken place. We did not present figures for T0 that took place 3 to 6 weeks after surgery. At this date, employment status was not in the focus of attention because the women were absorbed by post-surgical difficulties and follow-up treatment.

**Table 3:** Breast cancer patients: employment status by survey wave displayed for women who participated in all survey waves; categories are mutually exclusive

	Prior to survey	t1	t2	t3
Employed/self-employed	372/100%	190/51.07%	279/75.0%	265/71.07%
Sick leave	0	132/38.48%	40/10.75%	0
Not employed	0	6/1.61%	15/4.03%	10/2.69%
Gradual reintegration to work	0	37/9.94%	23/6.18%	0
Officially registered as unemployed	0	5/1.34%	8/2.15%	12/3.23%
Retired	0	2/0.54%	7/1.88%	84/22.66%
Missing data	0	0	0	1/0.35%
Sum	372/100%	372/100%	372/100%	372/100%

### *Regressions and supplementary analyses for breast cancer survivors (*Table 4*)*

**Table 4:** Results of regression analyses with effects of disease-related, therapy-related, and occupation-related factors on return to work after breast cancer surgery; confidence bounds based on 50 bootstrap samples (*OR*, odds ratio)

	**Third survey (t2), 12 months after surgery**			**Fourth survey (t3), 4–6 years after surgery**		
	**Disease- and therapy- related factors**			**Disease- and therapy- related factors**		
	**OR**	***p***	**95% CI**	**OR**	***p***	**95% CI**
Tumour stage T0 T1 T2 T3/T4	1 (ref.) 0.62 0.59 0.29	– 0.07 0.22 0.06	– 0.41–0.93 0.18–1.97 0.05–1.56	1 (ref.) 0.88 0.54 0.16	– 0.52 0.22 0.01	– 0.60–1.28 0.20–1.43 0.04–0.58
Antihormone therapy None Yes	1 (ref.) 0.80	– 0.35	– 0.50–1.29	1 (ref.) 0.80	– 0.27	– 0.54–1.19
Age (in years)	1.01	0.23	0.99–1.05	0.83	< 0.001	0.79–0.88
Constant	1.372	0.73	0.23–8.14	119,341	< 0.001	3612–3,492,168
	Oncological rehabilitation			Oncological rehabilitation		
	OR	***p***	95% CI	OR	***p***	95% CI
Outpatient rehabilitation No Yes	1 (ref.) 0.47	– 0.02	– 0.25–0.89	1 (ref.) 0.95	– 0.86	– 0.55–1.64
Age (in years)	1.023	0.156	99–1.06	0.836	< 0.001	0.776–0.901
Constant	1.51	0.65	0.25–0.07	77,241	< 0.001	1065–5600576
	Work-related factors			Work-related factors		
	OR	***p***	95% CI	OR	***p***	95% CI
Occupational autonomy 1. Low level 2. Some autonomy 3. Intermediate level 4. Elevated level 5. High level	1 (ref.) 1.31 1.44 0.84 0.79	– 0.67 0.52 0.75 0.75	– 0.49–4.44 0.47–4.36 0.28–2.51 0.18–4.41	1 (ref.) 1.35 1.57 1.30 4.56	– 0.58 0.23 0.57 0.04	– 0.46–3.99 0.75–3.29 0.52–3.26 1.10–18.81
Effort-reward imbalance	0.13	< 0.01	0.06–0.31	0.64	0.31	0.28–1.50
Age (in years)	1.01	0.49	0.98–1.05	0.839	< 0.001	0.772–0.913
Constant	11.43	0.06	0.90–144.69	61,551	< 0.001	518–7,305,999
	Educational level			Educational level		
	OR	***p***	95% CI	OR	***p***	95% CI
Education 8/9 years of school 10 years of school 12/13 years of school Other	1 (ref) 2.40 1.30 0.75	– 0.06 0.57 0.68	– 1.10–5.24 0.56–3.00 0.22–2.55	1 (ref) 2.25 2.23 1.73	– 0.03 0.03 0.42	– 1.10–4.63 1.08–4.63 0.46–6.51
Age (in years)	1.018	0.37	0.986–1.052	0.842	0.002	0.753–0.940
Constant	0.70	0.73	0.09–5.52	25,609	0.004	26–2,470,000

It has again to be noted that Table 4 depicts four separate lines of analysis, i.e. one for effects of disease- and therapy-related factors on return to work, one for effects of oncological rehabilitation, one for effects of work-related factors, and one for effects of educational level. Analyses with all these factors taken together were not possible due to insufficient case numbers.

For t2, 1 year after diagnosis, the odds ratios of the different *tumour stages* were decreasing. The more severe the disease, the lower the (statistically speaking) ‘chance’ for a return to work, but all effects were not consistently statistically significant. At T3, odds ratios also decreased with disease severity, but the effect for the category indicating the severest tumour stage included turned out as statistically significant. In both cases, *antihormone therapy* had no significant effects on return to work.

Inpatient oncological *rehabilitation* was associated with a lower ‘chance’ for return to work at t2, but not at t3, where it had no effects. To find out whether utilisation and disease stage were associated, a supplementary analysis was performed, and the cross-tabulation-based analysis revealed that this was not the case (*χ*^2^(2) = 0.63; *p* = 0.73). Participation and employment status turned out as being associated in the way that self-employed women were less likely to use inpatient rehabilitation than employed ones (*χ*^2^(1) = 24.67; *p* < 0.001).

At t2, *occupational autonomy* measured as before surgery had no effect on return to work, while only a high level of autonomy had an interpretable effect on employment at t3. In contrast, *effort-reward imbalance* obtained a significant effect at t2, but not at t3.

Effects of *educational level* were statistically insignificant for t2, but the figures for t3 are indicating that women with higher than basic education were more likely to return to work.

## Discussion

Our study compared breast cancer survivors with a multivariately drawn comparison group from the general population in order to examine disease-related exits from occupational life 4–6 years after primary surgery. The comparisons yielded a lower employment rate among breast cancer survivors. Both cohorts included a number of women who had reached retirement age at t3, but among breast cancer survivors, the rate of employed women was lower, thus pointing towards a higher rate of early retirement. Patients may have preferred to retire over being employed or being officially unemployed. By estimating the aftermaths of breast cancer, an increased likelihood of exit from economically active life occurred in addition to the immediate disease-related burden and subsequent impairment.

Although among breast cancer survivors the proportion of retired women was higher than in the comparison sample, return to work was rather the rule than the exception. This may have emerged because occupational work is considered an element of normal life and/or a material necessity. The high proportion of economically active women might lead to the conclusion that 4 to 6 years after surgery life may have been turned back to normal, but this is a view from outside that may underestimate the psychological consequences of the disease. In a companion paper, we have shown that many breast cancer survivors were impaired by long-lasting aftermaths, particularly due to medication with antihormonal therapy [36]. These side effects are affecting several dimensions of social activities as well as having psychological consequences such as anxiety and depression. Thus, if long-term consequences of breast cancer are considered, it is inadequate to assume consistency of psychological reactions, social life, and the job as they may vary independently.

Looking at the regression analyses, 12 months after surgery tumour stage and antihormone therapy did not have effects on return to work. At this time, aftermaths of surgery and aftercare can still be felt, and radiation and chemotherapy may still be carried on or may have ended not long ago. Within this context, occupation-related consequences may not have been in the focus of attention. Four to 6 years later, the situation has changed as the immediate aftermaths of primary treatment may have been overcome, but this may not apply to women with more advanced stages of their disease. Although quality of life under antihormonal therapy is impaired in several ways (e.g. sleep disturbances, fatigue, depression, anxiety, multimorbidity) [5, 10, 19], it appears that in the long run this did not have effects on return to work.

Oncological rehabilitation is offered by the German Pension Insurance as a supplementary part of cancer care for preventing disease-related premature retirement. In earlier studies, it was shown that this goal could not be achieved completely [22, 40], and it remains to be discussed why this was not the case. We have shown that tumour stage did not explain participation in the workforce. In an earlier study, the share of patients using oncological rehabilitation was lower among self-employed women. For this group, sickness absence leads to loss of income, and most of them are privately insured where rehabilitation is usually not included and has to be paid out of the pocket [22]. In our study, this finding was reproduced, but only 25 women in the patient sample were self-employed. Thus, further reasons for lower participation remain unexplained if only our data are considered. In an earlier mixed methods study, standardised questions were combined with open-ended responses. It turned out that the most frequently mentioned reasons for non-participation were the strong will to return back to normal as soon as possible, to avoid contact with patients with the same disease, and to resume work as distraction from the disease [8]. Taken together, the reasons for refraining from oncological rehabilitation appeared as subjective and not specific to defined social groups. Characteristics of the workplace have been reported to be relevant for returning to work. In our analyses, we have considered two structural aspects. One year after surgery, occupational autonomy turned out as irrelevant for return to work, and we assume that this may have been too early, because disease-related issues may still have been in the focus of attention. This interpretation does not appear as fully satisfying because effort-reward imbalance as the positive trade-off of return to work turned out as a positive aspect. This trade-off may come as a positive appreciation by employers and colleagues at the workplace, but again, we could not develop this aspect further. Then, 4 to 6 years after surgery, occupational autonomy had a marked effect on return to work, but only for women who reported a high degree of autonomy at work before diagnosis. Those who were self-employed were more likely to hold jobs high autonomy, but this did not clearly translate into a higher likelihood of return to work. Besides these rather structural aspects, return to work has repeatedly been reported to being dependent on the support of employers and/or colleagues [14, 20]. Specifically, this refers to their willingness to tolerate sickness absence, lower work performance, and to give support beyond the tangible aspects of work. Such aspects are specific to workplaces and not systematic; thus, they may not be depicted by structural properties of work. In earlier studies, educational level was associated with a higher likelihood of return to work. Our results are pointing to a more differentiated consideration of this finding as educational level was not related with return to work at 12 months after surgery, but this had changed 4 to 6 years later. It has to be discussed what is being measured by assessing education. Entering educational level and occupational autonomy in the same regression equation did not lead to interpretable results because both are correlated. The magnitude was not high enough to cause multicollinearity problems, but high enough to keep them separate in regression analyses. Nevertheless, it is justified to conclude that both are associated with return to work 4 to 6 months after surgery.

Finally, the role of age needs to be discussed. Since 2012, retirement age in Germany is elevated to 67 years in monthly steps until the year 2031. Thus, a rather trivial interpretation of the age effect is that exit from occupational life becomes more likely with age due to having reached regular retirement age. For all women with breast cancer who have not retired before, early retirement is more attractive from a financial point of view, and also disease-related and not disease-related multimorbidity may play a role. Thus, age-related effects may emerge in two ways.

## Limitations

Although our study was based on a relatively large number of patients, the size of our study population did not permit to include further potentially relevant factors that may have effects on return to work. Potential influencing factors are the meaning of work, the attachment to work, emotional distress, and the way patients are coping with their disease [14, 25]. Empirically, we had to divide the types of data into blocks of variables that could be analysed in regression analyses. This approach had also been necessary because some indicators were correlated so that the estimation of their effects in one regression equation might have caused undesirable outcomes. This applies, e.g. to occupational autonomy and educational level that have a rank order correlation of rho = 0.54. This is below the threshold where multicollinearity can be considered a statistical problem, but nevertheless there is enough shared variance; thus, it was advisable not to estimate their effects in the same analysis. The issue of sufficient case numbers has also impeded the inclusion of factors outside the structural level that have been reported to having effects, i.e. colleagues’ and employers’ consideration for patients’ short- and long-term impairments in order to facilitate return to work at microlevel [20]. The same applies to patient-related psychological and family-related factors [25] as well as different pathways of treatment [27].

To summarise, we conclude that, against the backdrop of high rates of economically active breast cancer survivors, the rate of women retiring prematurely was higher than in a comparable general population sample. The effects of factors assumed to govern return to work are dependent on the distance between surgery and measurement. Structural factors have become effective long after aftertreatment having ended.

## Data Availability

The data are available from the authors upon reasonable request, because patient data protection guidelines apply that do not permit to publish data directly. As the research group is not the owner of the SOEP- general population data where the contol group was drawn from, requests concerning the SOEP should be addressed to the SOEP-research data center: https://www.diw.de/de/diw_01.c.601584.de/datenzugang.html The STATA- DO-files for drawing the control group is available from the first author (SG).
